# A data‐driven search for semen‐related phenotypes in conception delay

**DOI:** 10.1111/andr.12288

**Published:** 2016-10-28

**Authors:** C. J. Patel, R. Sundaram, G. M. Buck Louis

**Affiliations:** ^1^ Department of Biomedical Informatics Harvard Medical School Harvard University Boston MA USA; ^2^ Division of Intramural Population Health Research Biostatistics and Bioinformatics Branch Eunice Kennedy Shriver National Institute for Child Health and Human Development The National Institutes of Health Rockville MD USA; ^3^ Division of Intramural Population Health Research Office of the Director Eunice Kennedy Shriver National Institute for Child Health and Human Development The National Institutes of Health Rockville MD USA

**Keywords:** conception delay, fecundity, semen analysis, sperm quality parameters

## Abstract

Sperm count, morphology, and motility have been reported to be predictive of pregnancy, although with equivocal basis prompting some authors to question the prognostic value of semen analysis. To assess the utility of including semen quality data in predicting conception delay or requiring >6 cycles to become pregnant (referred to as conception delay), we utilized novel data‐driven analytic techniques in a pre‐conception cohort of couples prospectively followed up for time‐to‐pregnancy. The study cohort comprised 402 (80%) male partners who provided semen samples and had time‐to‐pregnancy information. Female partners used home pregnancy tests and recorded results in daily journals. Odds ratios (OR), false discovery rates, and 95% confidence intervals (CIs) for conception delay (time‐to‐pregnancy > 6 cycles) were estimated for 40 semen quality phenotypes comprising 35 semen quality endpoints and 5 closely related fecundity determinants (body mass index, time of contraception, lipids, cotinine and seminal white blood cells). Both traditional and strict sperm phenotype measures were associated with lower odds of conception delay. Specifically, for an increase in percent morphologically normal spermatozoa using traditional methods, we observed a 40% decrease in conception delay (OR = 0.6, 95% CI = 0.50, 0.81; *p* = 0.0003). Similarly, for an increase in strict criteria, we observed a 30% decrease in odds for conception delay (OR = 0.7, 95% CI = 0.52, 0.83; *p* = 0.001). On the other hand, an increase in percent coiled tail spermatozoa was associated with a 40% increase in the odds for conception delay (OR = 1.4, 95% CI = 1.12, 1.75; *p* = 0.003). However, our findings suggest that semen phenotypes have little predictive value of conception delay (area under the curve of 73%). In a multivariate model containing significant semen factors and traditional risk factors (i.e. age, body mass index, cotinine and ever having fathered a pregnancy), there was a modest improvement in prediction of conception delay (16% increase in area under the curve, *p* < 0.0002).

## Introduction

Semen analysis is commonly used to evaluate male fecundity, defined as the biological capacity of men for reproduction irrespective of pregnancy intentions. Couples trying unsuccessfully for pregnancy after 6 or 12 months may undergo a clinical semen analysis, as it remains the standard for assessing male fecundity and related impairments (ASRM, 2006). While key components of semen analysis such as sperm concentration, motility and morphology are reported to be capable of classifying men by fertility (birth) potential (Guzick *et al*., 2001), established cut points for these endpoints still result in considerable misclassification. For example, men below norms may still go on to father pregnancies resulting in a live birth, while men with values within ranges may be unable to do so. The World Health Organization publishes reference values for semen parameters relative to fertility (Cooper *et al*., 2010; WHO, 2010). However, the predictive value of these reference values has long been debated for the simple reason that no single or set of semen parameters is highly predictive of male fertility (Wang *et al*., 1988; Niederberger, 2011; ASRM, 2013). These findings have prompted some researchers to encourage development of new biomolecular or methodological (e.g. sperm energy index or genomic) approaches beyond functional testing to better predict male fecundity and fertility (Isobe, 2012; Kovac *et al*., 2013).

Despite the need for prospective couple‐based study designs capable of estimating the predictive value of semen phenotype endpoints for couple fecundity [e.g. time‐to‐pregnancy (TTP)] or fertility (e.g. live births), only a few such studies exist across the globe. Such studies recruit couples upon discontinuing contraception so they can be followed through a year of trying for pregnancy, with the male partner providing baseline and, possibly, additional semen samples. The rarity of such studies has been described (Buck Louis *et al*., 2004) and is likely one explanation for our inability to develop methods for the sensitive and specific prediction of male fecundity and related impairments based upon semen analysis results. In fact, we are aware of only two previous prospective cohort studies that enrolled and followed couples to assess semen quality in relation to couple fecundity, as measured by TTP (Bonde *et al*., 1988; Zinaman *et al*., 2000). In the first such study, no significant associations were observed between semen volume and motility; however, sperm concentration up to 40 × 10^6^/mL and percent normal morphology (10–60%) were independently associated with the probability of pregnancy (Bonde *et al*. 1998), as was sperm count and percentage of normal spermatozoa in another study (Zinaman *et al*., 2000) even considering couples’ ages, body mass indices and cigarette smoking histories.

In an attempt to move the field forward, we utilized multivariate data techniques inspired by the exposome research paradigm (Wild, 2012; Buck Louis *et al*., 2013) to investigate individual semen quality endpoints in association with conception delay (or, time‐to‐pregnancy greater than six cycles). Prioritization and/or hypothesis generation research aimed at delineating the prognostic role of semen phenotypes for male fecundity may benefit from the use of emerging methods capable of systematically analysing many factors to identify environmental exposures and phenotypes associated with disease (Patel & Ioannidis, 2014). Examples of analogous data‐driven investigations included searches of environmental or behavioral factors associated with type 2 diabetes (Patel *et al*., 2010), serum lipids (Patel *et al*., 2012), blood pressure (Tzoulaki *et al*., 2012), endometrial cancer (Merritt *et al*., 2015), all‐cause mortality (Patel *et al*., 2014), family income (Patel *et al*., 2015) and pre‐term birth (Patel *et al*., 2013).

In this study, we aimed to investigate the role of the predictive value of semen phenotypes in the prospective study of a clinically relevant outcome – conception delay. This overall aim is important given the debate surrounding the clinical utility of semen phenotypes in male fecundity. Furthermore, we aimed to determine the predictive capability of the identified semen phenotypes in conception delay. As such, we are not focusing on environmental chemicals but on phenotypes previously reported to be associated with fecundity that are used in clinical practice as a prediction tool. Specifically, we sought to estimate whether inclusion of semen phenotypes in a predictive model improves clinical prediction of fecundity impairments, such as conception delays, relative to more traditional type models without semen characteristics.

Two other studies reported little to no association between semen phenotypes and TTP (Andersen *et al*., 2002; Buck Louis *et al*., 2014). In fact, we previously assessed the association between each semen phenotype and TTP (Buck Louis *et al*., 2014) in the same study cohort investigated here. In our previous paper, we reported that most semen phenotypes were not significantly associated with TTP after adjusting for male's age and/or BMI. In contrast to our previous work where semen phenotypes were only assessed in TTP individually, we utilized the aforementioned data‐driven techniques to search for semen phenotypes predictive of conception delay (as measured by a time‐to‐pregnancy >6 cycles). Secondly and critically, we assess the predictive value (and clinical relevance), if any, of semen phenotypes in conception delay. This predictive model is compared with a multivariate model of established and conventional risk factors, such as age and smoking status.

## Materials and Methods

### Design and study population

A prospective cohort design with pre‐conception recruitment of couples was used to assess semen quality and impaired fecundity, defined as requiring >6 prospectively observed menstrual cycles to become pregnant. The study cohort was restricted to 402 male partners of couples participating in the LIFE Study who provided semen samples and for whom a TTP was prospectively observed. Complete description of the LIFE Study methods is provided elsewhere (Buck Louis *et al*., 2014).

### Data and semen collection

Male partners were interviewed at baseline about lifestyle and medical history inclusive of reproductive history. Trained research assistants weighed men and measured their height using standardized methods and calibrated scales and measuring tapes for the calculation of body mass index (BMI; weight in kg/height in m^2^). A blood sample was obtained for the measurement of serum cotinine as a marker of active cigarette smoking. Shortly after enrolment, men collected a baseline semen sample. Men were instructed to collect the sample following 2 days of abstinence via masturbation without the use of any lubricants or condoms. At home collection kits were provided and included an insulated shipping container (Hamilton Research, Beverly, MA, USA) to maintain sperm integrity, a glass specimen jar to which a temperature data logger (I‐Button; Maxim Integrated, Jan Jose, CA, USA) was attached to ensure temperature stability from collection through laboratory analysis, a sperm migration straw filled with hyaluronic acid and plugged at one end to capture motile spermatozoa at collection, and packing materials (Vitrotubes #3520; VitroCom Inc., Mt. Lakes, NJ, USA). The at home collection protocol has been described elsewhere (Royster *et al*., 2000). After collection, men recorded any spillage and date of last ejaculation on labels and placed ice packs in containers prior to returning via next day delivery. All samples were received in good condition and analysed within 24 h, recognizing our motility measures reflect next day motility. All human subjects provided approval to use their information for research to all participating institutions.

### Andrology analysis

Semen samples were analysed by one highly experienced andrology laboratory using established standard operating procedures inclusive of ongoing quality assurance and control (Buck Louis *et al*., 2014). Briefly, sperm motility was assessed using the HTM‐IVOS (Hamilton Thorne, Beverly, MA, USA) computer‐assisted semen analysis system, sperm concentration using the IVOS system and the IDENT stain (Hamilton Thorne, Beverly, MA, USA), sperm viability by the hypo‐osmotic swelling assay, and sperm morphometry using the IVOS METRIX system (Hamilton Thorne, Beverly, MA, USA). A contracting laboratory assessed sperm morphology using both traditional and strict morphology classifications (WHO, 2010; Rothmann *et al*., 2013). SCSA was measured on the first specimen (Evenson *et al*., 2002) and using a Coulter Epics Elite Flow Cytometer and software (SCSA diagnostics, Brookings, SD, USA). We assessed 35 semen characteristics, including five general characteristics (volume, straw distance, sperm concentration, total count and hypo‐osmotic swollen), eight motility measures (average path, straight and curvilinear velocity, amplitude head displacement, beat cross frequency, % motility, % straight and % linear movement), six sperm head measures (length, area, width, perimeter, % elongation factor and % acrosome area of head), 12 morphology measures (% normal – strict and WHO criteria; and % amorphous, round, pyriform, bicephalic, tapered, megalo/micro head, neck/midpiece abnormalities, coiled/other tail abnormalities, cytoplasmic droplet and immature sperm), and two sperm chromatin stability measures (% DNA fragmentation index and high fragmentation stainability). Of note, four semen parameters are composites of other measures, including sperm concentration that is derived from total sperm count divided by volume, and sperm head area, perimeter and elongation factor that are derived from sperm head length and width measurements. The laboratory's standard operating procedures are inclusive of ongoing quality control. The integrity of all semen samples upon arrival at the laboratory was assessed and all were found acceptable. Before analysis, all data were inspected for laboratory drift by batch and time with no significant differences observed (see Figure S1).

We also included five non‐semen phenotypes that may affect semen quality or impaired fecundity, including body mass index, serum cotinine, seminal white blood cell count, serum lipids and the number of months the couple was off contraception prior to enrolment. Collectively, we considered 40 variables comprising the 35 semen endpoints and 5 other related determinants of male fecundity.

### Statistical analysis

**Figure 1:**
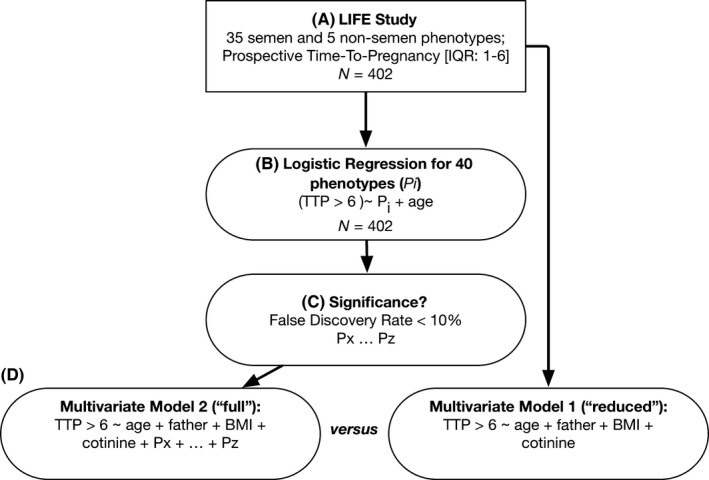
Analytical procedure to search for semen endpoints associated with conception delay, as measured by a time‐to‐pregnancy (TTP) >6 prospectively observed menstrual cycles. (A) The study cohort includes 402 males with prospectively observed TTP and 35 semen and 5 non‐semen phenotypes. (B) We performed age‐adjusted logistic regression to assess each of the 40 phenotypes (individual phenotypes denoted by Pi) with impaired fecundity or a TTP > 6 cycles. (C) Significant phenotypes are those achieving a false discovery rate (FDR) < 10%. (D) We compared two multivariate logistic regression models to determine if the inclusion of semen phenotypes improved prediction of impaired fecundity.

The statistical analysis was carried out in four steps as illustrated in Fig. 1 using an extension an ‘environment wide association study’ (EWAS) techniques (Patel & Ioannidis, 2014; Patel *et al*., 2014). First, we assessed each of the 40 phenotypes (35 semen endpoints and 5 non‐semen phenotypes) with conception delay, as measured by a TTP > 6 cycles, adjusted by age, to identify significant predictors (Fig. 1B). All variables were rescaled by their standard deviations to facilitate comparison of the magnitude of observed associations. Next, we used the Benjamini–Hochberg False Discovery Rate (FDR; Benjamini & Hochberg, 1995) with an a priori established FDR of ≤10% (*p*‐value of 0.01) to denote statistical significance (Fig. 1C). This step minimizes Type 1 error arising from chance findings given the number of statistical tests being made (40).

In step D (Fig. 1D), we compared two modelling scenarios to empirically assess the added utility of including semen endpoints above other possible fecundity risk factors (subsequently referred to as ‘traditional risk factors’) defined in the literature, including (i) age, (ii) ever having previously fathered a pregnancy, (iii) BMI and (iv) serum cotinine (Jensen *et al*., 2004; Auger & Jouannet, 2005; Ramlau‐Hansen *et al*., 2007; Sadeu *et al*., 2010; Rybar *et al*., 2011). This step considers how the FDR identified factors concurrently are predictive conception delay. Specifically, we compared the full model that included the four most significant phenotypes (FDR ≤ 10%) plus male risk factors (age, ever fathered a pregnancy, BMI and cotinine) to the model with only risk factors (age, ever having fathered a pregnancy, BMI and cotinine). Using analysis of variance (anova), we assessed the differences in the predictive capability of each model. We also used area under the receiver‐operator curve (AUC) to quantify the accuracy of the full model vs. the risk factor only model. By modelling multiple semen phenotypes in a single predictive model, their inter‐correlation is explicitly considered. In other words, factors that are independently associated with conception delay (or time‐to‐pregnancy >6 cycles) will remain significant in the model even after adjusting for other correlated variables.

Lastly, to illustrate the relatedness of semen quality and risk factors, we estimated pairwise Pearson correlations and illustrate them in a heatmap using a hierarchical clustering algorithm (Johnson & Wichern, 1992), as previously described (Patel *et al*., 2014). The figure shows how correlated variables are to one another; for example, adjacent variables on the heatmap are more correlated than those that are not adjacent.

## Results

Couples whose male partners were older or who had higher serum cotinine concentrations indicative of active smoking and who never fathered a pregnancy were significantly more likely to experience impaired fecundity, as measured by a TTP > 6 cycles, in comparison to their respective counterparts (Table 1). We did not observe a significant difference in body mass index and serum lipid concentration between males who had impaired fecundity vs. those who did not.

**Table 1:** Mean comparison of men by impaired fecundity status for the traditional risk factors (*n* = 402)

Characteristic	TTP ≤ 6 cycles Mean (SE) (*n* = 302)	TTP > 6 cycles Mean (SE) (*n* = 100)	*p*‐Value
Age (years)	31.2 (0.3)	33.3 (0.5)	0.0005
Body mass index (kg/m^2^)	29.8 (0.3)	29.2 (0.6)	0.55
Serum lipids (ng/g)	726.8 (12.0)	713.5 (22.0)	0.62
Serum cotinine (ng/mL)	40.9 (6.7)	79.9 (17.3)	0.04
Fathered previous pregnancies (#)	1.3 (0.04)	1.6 (0.13)	0.07

SE, standard error; TTP, time‐to‐pregnancy or the number of prospectively observed menstrual cycles required to become pregnant. Significance was assessed using the chi‐squared test for categorical and the *t*‐test for continuous variables.

**Figure 2:**
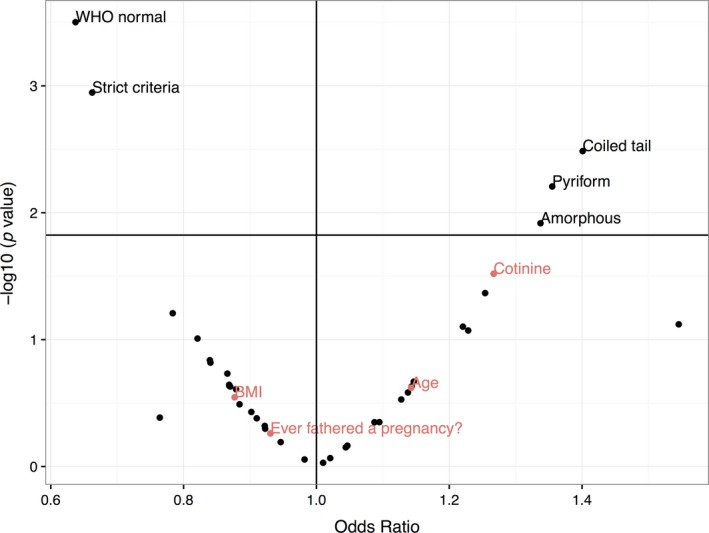
Illustration of the significant odds ratios for conception delay, as measured by at time‐to‐pregnancy >6 prospectively observed menstrual cycles, and statistical significance. Black horizontal line denotes false discovery rate (FDR) 10%. Semen phenotypes with FDR < 10% are annotated. Risk factors seen in red.

In our initial exploration of the associations between 40 semen phenotypes with impaired fecundity adjusting for age (Table S1), 5 (13% of 40) were found to be significant when using an FDR ≤10% (*p* < 0.01) (Table 2). These findings included: percent coiled tail (OR: 1.4; 95% CI: 1.12, 1.75; *p* = 0.003), percent pyriform (OR: 1.36; 95% CI: 1.09, 1.68; *p* = 0.01) and percent amorphous (OR: 1.34; 95% CI: 1.07, 1.68; *p* = 0.01) spermatozoa. These estimates reflected a 40, 36 and 34% higher odds of impaired fecundity, associated with sperm tail and head abnormalities, respectively. Conversely, a reduced odds of conception delay was observed for two other semen phenotypes. An increasing percent of morphologically normal spermatozoa using either the WHO (OR: 0.64; 95% CI: 0.50, 0.81; *p* < 0.001) or strict (OR: 0.66; 95% CI: 0.52, 0.85; *p* < 0.001) criteria was associated with a lower odds of conception delay. Figure 2 illustrates the semen phenotypes found associated with conception delay along with four traditional risk factors (i.e. age, ever fathered a pregnancy, BMI and cotinine) and summarizes them by magnitude and direction of their odds ratios. This visualization facilitates comparison of the association sizes for the study variables.

**Table 2:** Semen phenotypes and odds of impaired (TTP > 6 cycles) fecundity

Semen phenotype	OR (95% CI)	*p*‐Value	FDR
% Normal morphology – WHO criteria	0.64 (0.50, 0.81)	0.0003	0.01
% Normal morphology – strict criteria	0.66 (0.52, 0.85)	0.0011	0.02
% Coiled tail	1.40 (1.12, 1.75)	0.0033	0.04
% Pyriform spermatazoa	1.36 (1.09, 1.68)	0.01	0.06
% Amorphous spermatozoa	1.34 (1.07, 1.68)	0.01	0.10

CI, 95% confidence interval; FDR, false discovery rate; OR, odds ratio; TTP, time‐to‐pregnancy. Of the 40 semen phenotypes assessed, five were significant (FDR < 10%). All semen phenotypes are modelled per 1 standard deviation change and are age adjusted.

**Table 3:** Comparison of semen phenotype and risk factor only models for predicting impaired fecundity – multivariate logistic regression models

Variable	Full model –semen phenotypes and risk factors		Reduced model – risk factors only	
	OR (95% CI)	*p*‐Value	OR (95% CI)	*p*‐Value
Age	1.66 (1.29, 2.15)	9 × 10^−5^	1.67 (1.06, 1.17)	3 × 10^−5^
Ever fathered a pregnancy	0.45 (0.26, 0.75)	0.002	0.44 (0.52, 0.98)	0.04
Body mass index	0.87 (0.67, 1.11)	0.25	0.93 (0.73, 1.17)	0.54
Serum cotinine	1.27 (1.01, 1.60)	0.04	1.28 (1.03, 1.59)	0.03
% Normal morphology – WHO criteria	1.23 (0.73, 2.12)	0.44	–	–
% Coiled tail	1.52 (1.09, 2.14)	0.02	–	–
% Pyriform spermatozoa	1.46 (1.08, 2.01)	0.01	–	–
% Amorphous spermatozoa	1.57 (1.07, 2.35)	0.03	–	–
Nagelkerke *R* ^2^ (AUC)	0.18 (0.73)		0.11 (0.63)	

AUC, area under the curve. Impaired fecundity denotes a prospectively observed time‐to‐pregnancy > 6 cycles. ‘Full model’ includes all semen phenotypes achieving a false discovery rate < 10% along with age, ever fathered a pregnancy, body mass index and serum cotinine. ‘Reduced model’ includes only risk factors. All factors were rescaled by their standard deviation for analysis except ever fathered a pregnancy (yes/no). Nagelkerke *R* ^2^ represents the difference in the explained variance for the two models.

**Figure 3:**
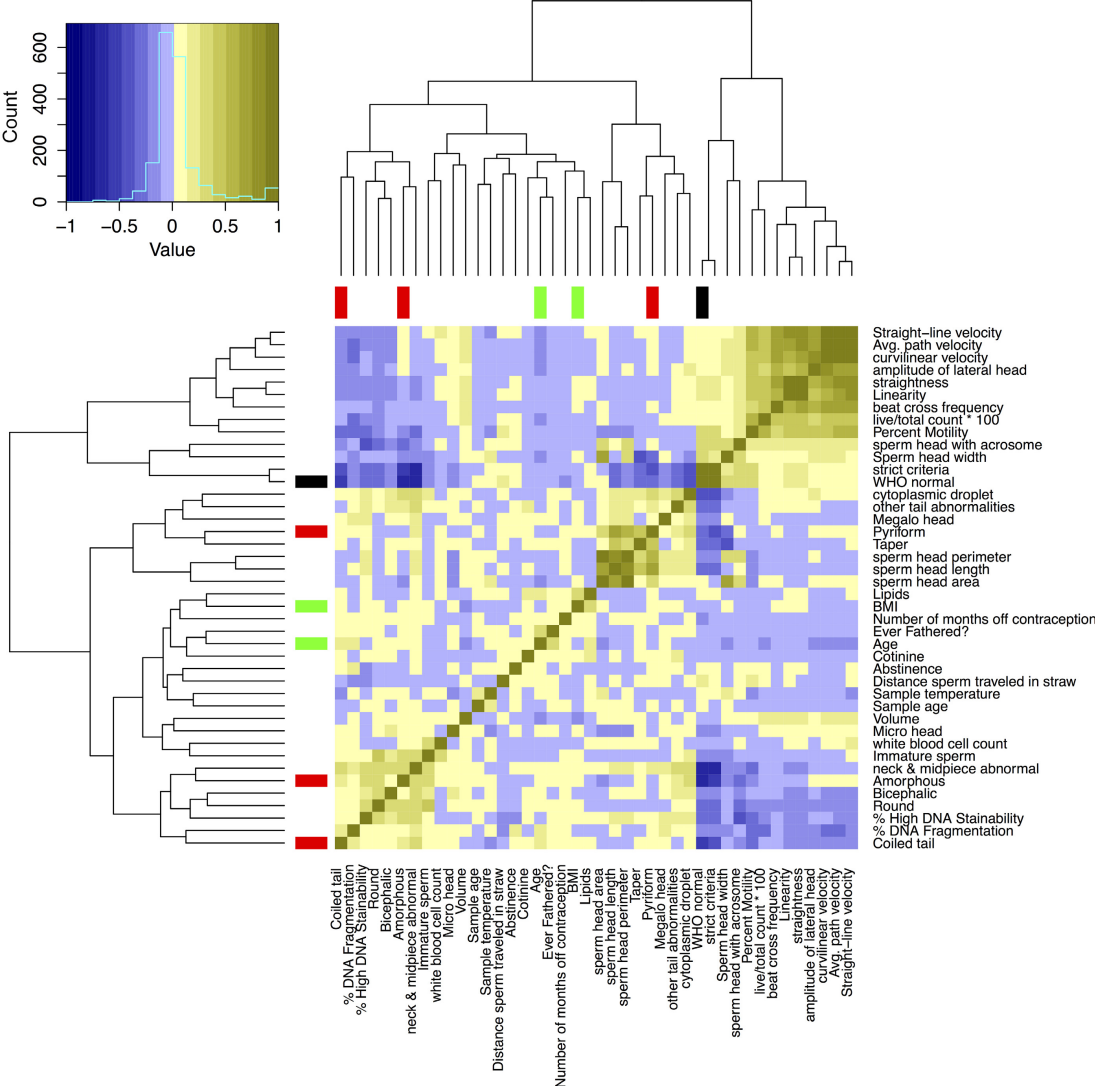
Pairwise correlation heatmap of 40 semen phenotypes and risk factors. Semen phenotypes with an false discovery rate (FDR) < 10% and an OR < 1.0 are seen in black (% strict criteria and WHO normal), while semen phenotypes with an FDR < 10% and an OR > 1.0 are seen in red (% pyriform, amorphous, and coiled tail). Risk factors are in green.

Many of the 40 phenotypes were correlated as illustrated in the heatmap (Fig 3), although most phenotypes exhibited low correlations with one another (median correlation of −0.002 and an interquartile range of −0.1 and 0.1). Still, some phenotypes were more highly correlated, viz., velocity, linear/straight sperm movement and percent motile spermatozoa (Fig. 3). Of interest, semen phenotypes that were inversely associated with impaired fecundity (normal morphology – strict and WHO criteria) were strongly correlated (Pearson ρ = 0.96, *p* < 10^−16^) with each other, while those that were positively associated were not.

When comparing the full (four significant semen phenotypes and traditional risk factors) and reduced (traditional risk factors) models in terms of predicting conception delay, the former model was empirically a better predictor (Table 3). Note that percent normal morphology based on strict criteria was not included in the full model, given that it was highly correlated with normal morphology based upon WHO criteria. The full and reduced models were significantly different (anova *p* = 0.0002 [Table 3]) and the full model was a marginally better predictor of conception delay or a time‐to‐pregnancy >6 cycles. Specifically, the AUC was greater for the full than reduced model (0.73 vs. 0.63, respectively [Table 3]). While the ORs remained relatively consistent across the risk factor and multivariate models, percent normal morphology (WHO criteria) lost significance (Table 3).

## Conclusions

To our knowledge, this is the first attempt to comprehensively search for and gauge the predictive power of semen phenotypes for predicting conception delay. Our study's aims were twofold: (i) to demonstrate the feasibility and utility of novel data‐driven techniques for assessing semen quality endpoints and human fecundity and (ii) to empirically determine if such techniques better predict conception delay, as defined by requiring >6 menstrual cycles to become pregnant, over traditional clinical risk factor approaches.

This analytic approach allows for the simultaneous analysis of a multitude of semen phenotypes and risk factors for predicting fecundity‐related outcomes, while accounting for the multiple comparisons. While the analyses conducted herein have been used to search for associations in chronic diseases and biomarkers such as serum lipids (Patel *et al*., 2012), we are not aware of any published work focusing on human fecundity or related impairments such as conception delay or pregnancy loss. To this end, we cannot interpret our findings in the context of earlier work, but data‐driven techniques can be extended to include emerging biomarkers of male fecundity, such as sperm function tests or in vitro capacitation tests (Wang & Swerdloff, 2014).

There are some limitations of our investigation. First, we did not aim to predict time‐to‐pregnancy and instead, attempted to predict a dichotomous outcome, conception delay (or time‐to‐pregnancy >6 cycles). We felt this choice was justified as conception delay has been empirically shown to be a very good marker of identifying which couples will need clinical evaluation and/or treatment. While there have only been a few studies that have followed couples from discontinuing contraception through 12 months of trying, the data demonstrate that 76–90% of couples who do become pregnant do so within the first 6 months of trying (Gnoth *et al*., 2003; Wang *et al*., 2003; Buck Louis *et al*., 2009, 2011). Moreover, clinical guidance suggests that couples seek care if not pregnant within 6 months of trying when the female partner is 35+ years of age.

Our findings do underscore the known importance of biology (i.e. age and previously fathered a pregnancy), lifestyle (i.e. cigarette smoking) and semen quality (i.e. sperm head and tail abnormalities) for predicting impaired fecundity or conception delay. Of particular note is the relatively similar observed magnitude for both age and sperm abnormalities relative to the odds of conception delay (or time‐to‐pregnancy > 6 cycles), and the importance of prior history (fathered a pregnancy) for predicting subsequent reproductive performance. Furthermore, while speculative, our findings suggest little added utility of including semen phenotypes for predicting which couples may experience delays in conceiving based upon semen characteristics. We note, on the other hand, that there is some evidence suggesting that morphologically abnormal spermatozoa may be associated with chromosomal abnormalities or alterations in chromatin packing and DNA fragmentation, which in turn may impact fertilization (Tang *et al*., 2010; Sivanarayana *et al*., 2012).

There are important study strengths that underlie these findings including our population‐based sampling approach that did not rely on men seeking clinical care, a high percent of men providing semen samples, in‐depth semen analysis of 35 endpoints, well measured risk factors, and prospectively measured time‐to‐pregnancy using home pregnancy test kits with demonstrated sensitivity for detecting 25 mIU/mL of human chorionic gonadotropin (hCG) and accurately used by women (Johnson *et al*., 2015). Still, important limitations need to be considered including our reliance on 24‐h motility measures in light of at home semen collection, the potential for chance findings and residual confounding given the study's observational design. Also, we opted to use analytical techniques for impaired fecundity (time‐to‐pregnancy > 6 cycles) rather than infertility (time‐to‐pregnancy > 12 cycles), given that most (68–90%) couples trying for pregnancy do so within 6 months as observed in prospective pregnancy studies with preconception enrolment and 12 months of follow‐up (Gnoth *et al*., 2003; Wang *et al*., 2003; Buck Louis *et al*., 2009, 2014). Also, couples may not wait 12 months before seeking clinical care. As such, the findings are relevant for couples experiencing delays becoming pregnant and not just ‘infertile’ couples. Semen phenotypes are one way of evaluating male and couple fecundity and fertility potential. The supporting literature is inconclusive with few leads prompting some investigators to question the continue utility of semen analysis (e.g. Niederberger, 2011).

As much of the past work has assessed each semen endpoint individually, we sought to answer a simple question – would using a data‐driven approach with all available semen quality data be predictive of impaired fecundity. Our findings suggest little to no clinical value in using sperm phenotypes as a predictor of conception delay. We note that our ‘expanded model’, a model with three sperm morphology phenotypes, improves prediction 16% above a model based on so‐called traditional risk factors. While an improvement, the receiver‐operator characteristic area under the curve was better than random chance (73%), but not high enough to be used as a prognostic.

Prediction may perhaps be improved by the analysis of a multitude of exposures or semen measures that characterize human populations. Moreover, these models can accommodate emerging biomarkers and provide an empirical estimate for improvement in prediction that might help advice clinical practice. With increasing concern about the impact of endocrine disrupting chemicals on male fecundity, particularly in light of a large fraction of male infertility attributed to such exposure (Hauser *et al*., 2015), coupled with evolving biomarkers of male fecundity (Isobe, 2012; Kovac *et al*., 2013), environment‐wide association study (EWAS) approaches offer options for identifying predictors of male fecundity and fertility. Novel approaches are needed if we are to understand the relatively high prevalence of male infertility in some populations (Louis *et al*., 2013), reported declines in sperm morphology (Rolland *et al*., 2013) and more generalized concerns about declining human fecundity (Skakkebaek *et al*., 2006; te Velde *et al*., 2010).

While our predictive model is modest, we hope our findings prompt other researchers to utilize these approaches in relation to other fecundity or fertility outcomes, and to, in the very least, corroborate our findings. Much of the focus of this paper is illustrative – to describe and demonstrate the application of data‐driven methods to investigate semen quality and fecundity. We believe the modest improvement in prediction supports evaluating approaches such as those presented in this study before abandoning semen analysis altogether to diagnose azoospermia.

## Disclosures

The authors have no financial interests that might benefit from this publication.

## Authors’ Contributions

Chirag J. Patel helped formulate the research question, designed and performed the statistical analyses, and drafted the paper. Germaine M. Buck Louis is the Principal Investigator for the LIFE Study and helped formulate this research question and in the writing of this paper. Rajeshwari Sundaram is the lead statistical investigator for the LIFE Study and assisted in the development of the analytic plan.

## Supporting information


**Figure S1.** Sperm phenotype quality assurance in the LIFE study across batch.
**Table S1.** Odds ratios, *p*‐values, false discovery rate for all 40 phenotypes.
