# Supplementary material for: A data‐driven search for semen‐related phenotypes in conception delay
Source: Andrology. 2016 Oct 28;5(1):95–102. doi: 10.1111/andr.12288 (PMC5164952; doi:10.1111/andr.12288)
Supplement: Supplementary file 1 — Figure S1. Sperm phenotype quality assurance in the LIFE study across batch. Table S1. Odds ratios, p‐values, false discovery rate for all 40 phenotypes. [file ANDR-5-95-s001.docx]

**A Data-driven Search for Semen-related Phenotypes of Conception Delay**

Supplementary File

Contents:

Supplementary Table 1. Odds ratios, p-values, false discovery rate for all 40 phenotypes.

Supplementary Figure 1. Sperm Phenotype Quality Assurance in the LIFE Study across batch.

Supplementary Table 1. Odds ratios, p-values, false discovery rate for all 40 phenotypes.

| **Description** | **OR [95% CI]** | **p-value** | **FDR** |
| --- | --- | --- | --- |
| WHO normal (1SD ) | 0.64 [0.50, 0.81] | 0.0003 | 0.01 |
| strict criteria (1SD ) | 0.66 [0.52, 0.85] | 0.0011 | 0.02 |
| Coiled tail (1SD ) | 1.40 [1.12, 1.75] | 0.0033 | 0.04 |
| Pyriform (1SD ) | 1.36 [1.09, 1.68] | 0.01 | 0.06 |
| Amorphous (1SD ) | 1.34 [1.07, 1.68] | 0.01 | 0.10 |
| Cotinine (Male, log-transformed and 1SD) | 1.27 [1.02, 1.57] | 0.03 | 0.20 |
| % DNA Fragmentation (1SD) | 1.25 [1.01, 1.56] | 0.04 | 0.25 |
| Percent Motility (1SD) | 0.78 [0.61, 1.01] | 0.06 | 0.31 |
| Immature sperm (1SD) | 1.55 [0.96, 2.50] | 0.08 | 0.31 |
| % High DNA Stainability (1SD) | 1.22 [0.98, 1.52] | 0.08 | 0.31 |
| sperm head length (1SD) | 1.23 [0.97, 1.55] | 0.08 | 0.31 |
| Sperm head width (1SD) | 0.82 [0.65, 1.04] | 0.10 | 0.33 |
| sperm head with acrosome (1SD) | 0.84 [0.66, 1.06] | 0.15 | 0.43 |
| Sample temperature (1SD) | 0.84 [0.66, 1.07] | 0.15 | 0.43 |
| straightness (1SD) | 0.87 [0.70, 1.07] | 0.19 | 0.45 |
| Round (1SD) | 1.15 [0.92, 1.42] | 0.21 | 0.45 |
| Straight-line velocity (1SD) | 0.87 [0.69, 1.09] | 0.23 | 0.45 |
| live/total count * 100 (1SD) | 0.87 [0.69, 1.09] | 0.23 | 0.45 |
| amplitude of lateral head (1SD) | 0.87 [0.69, 1.09] | 0.23 | 0.45 |
| Taper (1SD) | 1.14 [0.92, 1.43] | 0.23 | 0.45 |
| Number of months off contraception (1SD) | 1.14 [0.91, 1.43] | 0.24 | 0.45 |
| Linearity (1SD) | 0.88 [0.71, 1.09] | 0.25 | 0.45 |
| neck & midpiece abnormal (1SD) | 1.14 [0.91, 1.43] | 0.26 | 0.45 |
| Lipids (1SD) | 0.88 [0.69, 1.12] | 0.28 | 0.47 |
| cytoplasmic droplet (1SD) | 1.13 [0.90, 1.41] | 0.30 | 0.47 |
| Distance sperm traveled in straw (1SD) | 0.88 [0.69, 1.13] | 0.32 | 0.50 |
| Avg. path velocity (1SD) | 0.90 [0.72, 1.13] | 0.37 | 0.55 |
| white blood cell count (1SD) | 0.76 [0.40, 1.45] | 0.41 | 0.58 |
| curvilinear velocity (1SD) | 0.91 [0.72, 1.14] | 0.42 | 0.58 |
| sperm head perimeter (1SD) | 1.09 [0.87, 1.38] | 0.45 | 0.58 |
| Bicephalic (1SD) | 1.09 [0.88, 1.35] | 0.45 | 0.58 |
| beat cross frequency (1SD) | 0.92 [0.74, 1.15] | 0.48 | 0.60 |
| Volume (1SD) | 0.92 [0.73, 1.17] | 0.50 | 0.61 |
| BMI (1SD) | 0.93 [0.74, 1.18] | 0.55 | 0.64 |
| sperm head area (1SD) | 0.95 [0.75, 1.20] | 0.64 | 0.73 |
| other tail abnormalities (1SD) | 1.05 [0.84, 1.30] | 0.68 | 0.76 |
| Sample age (1SD) | 1.04 [0.83, 1.31] | 0.71 | 0.76 |
| Micro head (1SD) | 1.02 [0.81, 1.28] | 0.86 | 0.90 |
| Megalo head (1SD) | 0.98 [0.78, 1.24] | 0.88 | 0.90 |
| Abstinence (1SD) | 1.01 [0.79, 1.28] | 0.94 | 0.94 |

**Supplementary Figure 1. Sperm Phenotype Quality Assurance in the LIFE Study across batch.**

**
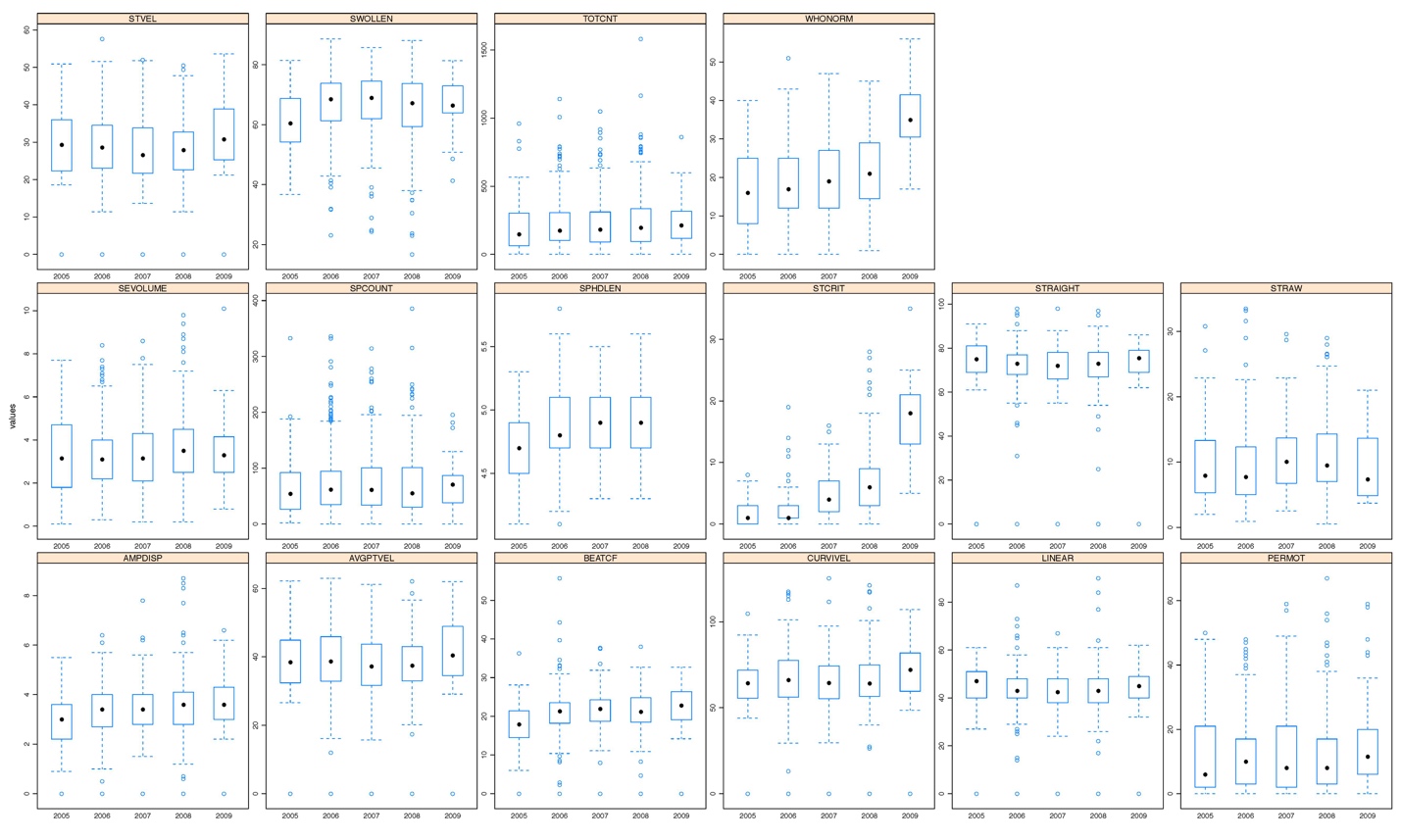
**
